# Academic performance and intelligence quotient of primary school children in Enugu

**DOI:** 10.11604/pamj.2020.36.129.22901

**Published:** 2020-06-25

**Authors:** Uzoamaka Chinenye Akubuilo, Kenechukwu Kosisochukwu Iloh, Justus Uchenna Onu, Adaeze Chikaodinaka Ayuk, Agozie Chukwunedum Ubesie, Anthony Nnaemeka Ikefuna

**Affiliations:** 1 Department of Paediatrics, University of Nigeria Teaching Hospital, Ituku-Ozalla, Enugu, Nigeria,; 2 Department of Paediatrics, College of Medicine, University of Nigeria, Enugu, Nigeria,; 3 Department of Mental Health, Nnamdi Azikiwe University, Awka, Anambra State, Nigeria

**Keywords:** Academic performance, intelligence quotient, primary, school

## Abstract

**Introduction:**

intellectual capacity measured as intelligence quotient (IQ) is one of the determinants of school performance of children. It influences academic achievement, future personal health, social well-being and therefore, is of public health significance. The objective of the study was to determine the intelligence quotient (IQ) and academic performance of primary school children in Enugu-East LGA.

**Methods:**

children who met the inclusion criteria were recruited from both public and private primary schools in the Local Government Area (LGA) using a proportionate multistage sampling technique. Academic performance was classified into high, average and low academic using past records of class assessment. Intelligence quotient was assessed using the Raven´s Standard Progressive Matrices (RSPM) and was grouped into optimal and suboptimal. A semi-structured questionnaire was used to obtain data such as-age, gender, socio-economic indices and family size of the study participants. Analysis was done with Statistical Package for Social Sciences (IBM-SPSS).

**Results:**

a total of 1,122 pupils aged 6 to 12 years were recruited. Optimal IQ and high academic performance were found in 54.0% and 58.8% of the study participants. Being from upper social class, in private school, and family size less than 4 were the significant determinants of high IQ and good academic performance (p<0.001).

**Conclusion:**

low socio-economic status, large family size and public school attendance impact negatively on IQ and academic performance. Hence, measures to curb large family sizes (i.e.>4 children) and improve the socio-economic status of families are needed environmental measures to improve intelligence and academic performance.

## Introduction

Intelligence is the aggregate or global capacity of an individual to act purposefully, to think rationally and to deal effectively with the environment [1]. General intellectual functioning (referred to as intelligence quotient) typically refers to one´s global or overall level of intelligence [2]. Intelligence quotient (IQ) is critical for independent participation in core activities such as education, self-care, and in later life, employment and living independently [3]. Measures of ‘Global IQ’ reflect an individual's overall ‘ability to understand complex ideas, to adapt effectively to the environment, to learn from experience, to engage in various forms of reasoning, [and] to overcome obstacles by taking thought’ [4]. Intelligence is affected by both environmental and biologic factors [5]. Intelligence and academic performance are different but interrelated concepts as intelligence is said to be one of the most important cognitive factors responsible for the variations in achievement scores [6].

Intellectual capacity (measured as intelligence quotient or IQ) is said to be one of the determinants of poor school performance of children [5]. Academic achievement influences future personal health and is, therefore, of significant public health concerns [6]. Good education has been linked to better jobs, higher income, and higher socio-economic status; while poor school performance with its attendant risk of school dropout results in future income reductions and thereby perpetuates the inter-generational cycle of poverty. Still, there are conflicting reports in the literature regarding the relationship between IQ and academic performance [7-10]. Several factors such as nutrition, education, socio-economic status, age, gender, school type and family size may affect IQ and academic performance [11, 12]. This study aimed to determine the intelligence quotient and academic performance of primary school children in Enugu-East LGA, the correlation between the two as well as their relationship with socio-demographic variables (age, gender, socio-economic status, family-size and school-type).

## Methods

This was a cross-sectional descriptive study involving school-aged children (6 to 12 years) in Enugu-East local government area of Enugu State. The study was carried out over a 3-month period from November 2017 to February 2018. Children who had attended same school for the three preceding terms, whose parents gave consent and the child assented to participate in the study if seven years and above were recruited into the study. Children who met the inclusion criteria were recruited from both public and private primary schools using a proportionate multistage sampling technique. In the first stage, simple random sampling by balloting was used to select one public and one private school from each political ward. In stage two, based on the total number of estimated pupils in each school, samples were allocated accordingly using the proportionate allocation method. With proportionate allocation method, the sample size of each school was proportionate to the population of the school. In stage three, the allocated sample size for each school was re-allocated proportionately across pupils in the different classes. In stage four, within each class, the pupils to be studied were selected randomly using a computer generated table of random numbers.

**Figure 1:**
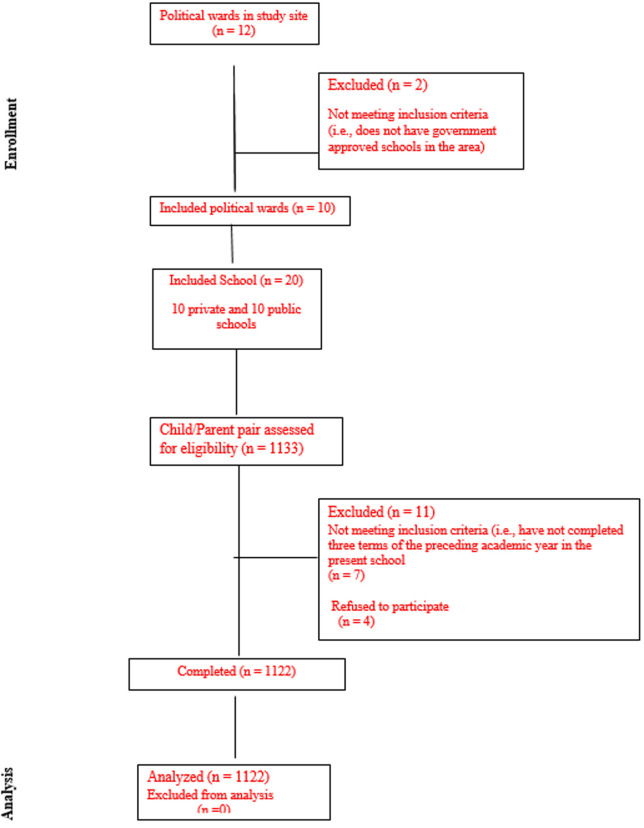
study flow chart (enrollment analysis)

In computing the required sample size for the study, we used the findings from Adedeji *et al*. [13] for the following reasons: first, they studied similar age group using the similar study instruments. Second, both studies were carried out in the same country. Based on their finding of the prevalence of sub-optimal IQ of 62.5%, we computed the required sample size by using this Figure 1 to substitute in the Araoye [14] formula for a population <10000 and arrived at a minimum sample size of 1098.

Intelligence quotient was assessed using the Raven´s Standard Progressive Matrices (RSPM). The Raven Standard Progressive Matrices Test (RSPM) is widely used as a general intelligence test in the world [15]. The Raven´s standardized progressive matrices (RSPM) was designed to measure a person´s ability to form perceptual relations and to reason by analogy independent of language and formal schooling, and may be used in persons ranging from six years to adulthood [16]. It has become one of the leading and most frequently used test of non-verbal or abstract reasoning and has been described as the paradigm test of non-verbal, abstract reasoning ability. This version has been effectively used and validated in Nigeria [9] and other African countries [17-20]. For ease of interpretation, RPM grades had been divided in two groups; optimal and suboptimal IQ [21].

The overall scores in percentage for each child in the three terminal examinations in four subjects (English language, Mathematics, Social studies and Primary science) during the completed academic year preceding the study period was documented and the average taken. This was used as an index of general academic performance. The performance was scored as high if ≥75 whereas 50-74% and <50% were scored as average and low respectively [22]. Those with a low overall score were regarded as having poor academic performance [22]. A semi-structured questionnaire was used to obtain data such as-age, gender, socio-economic indices and family size of the study participants. Data analyses were done using IBM-Statistical Package for Social Sciences (IBM-SPSS) version 20.0 (Chicago II). The normality of distribution of age, IQ raw scores and academic grades was checked using Shapiro-Wilk test. They were found to be normally distributed. The associations between intelligence quotient (categorized as optimal and sub-optimal)/academic performance (categorized as low, average, and high) and socio-demographic variables were tested using chi square. Binary and multinomial logistic regression was used to determine the strength of association between the socio-demographic variables that were significant at the univariate analysis and the intelligence quotients (optimal versus sub-optimal) and academic performance (low, average, and high), respectively. The relationship between IQ (optimal versus sub-optimal), age and academic performance (as continuous variables) was analyzed using independent t-test and effect size at 95% confidence interval was calculated. A p-value of less than 0.05 was regarded as significant.

**Ethics approval and consent to participate:** ethical approval was obtained from the Ethics and Research Committee of the University of Nigeria Teaching Hospital with protocol number NHREC/05/01/2008B-FWA00002458-IRB00002323 issued on the 7^th^of July, 2017. Informed consent was obtained from the parents of the children. The retrieved information was transferred into a private computer and passworded. Data was anonymized, and questionnaires had no names. Participation in the study was entirely voluntary, and no financial inducement whatsoever was involved. Participants were informed that voluntary withdrawal at any stage of interaction was guaranteed for them without any adverse effect. The datasets used and/or analyzed during the current study are available from the corresponding author on reasonable request.

## Results

**General characteristics of the study population:** a total of 1,122 pupils aged 6 to12 years were recruited from 10 public and 10 private schools in the local government. The mean age of the study participants was 9.0±1.7 years. The mean ages of the males and females were 9.0±1.7 and 9.0±1.6 years, respectively. The majority (53.6%) were females, from upper socio-economic class (39.1%), and in private school (53.9%). The prevalence of sub-optimal intelligence quotient among the study participants was 46.0%, whereas 58.8%, 35.3% and 5.9%, respectively had high, average and low academic performance (Table 1).

**Table 1:** the socio-demographic variables of the study participants

Variables	N (%)	Mean ± SD
**Age**		
Males		9.0±1.7
Females		9.0±1.6
**Gender**		
Males	521 (46.4)	
Females	601 (53.6)	
**Socioeconomic class**		
Upper	439 (39.1)	
Middle	300 (26.7)	
Lower	383 (34.1)	
**School type**		
Private	605 (53.9)	
Public	517 (46.1)	
**Intelligence Quotient**		
Optimal	606(54.0)	
Sub-optimal	516(46.0)	
**Academic Performance**		
High	660(58.8)	
Average	396(35.3)	
Low	66(5.9)	

**Relationship between socio-demographic variables and intelligence quotient:** there was a significant relationship between social class and intelligence quotient (X^2^=84.4, p<0.001), such that participants from upper socio-economic class were 2.9 times more likely than those from lower social class to have higher IQ [AOR, (95%CI); 2.9(1.8-3.6]. In addition, participants from private schools and those whose family size was less than 4 were significantly more likely to have higher IQ [AOR, (95% CI); 3.7(2.9-4.8) and 1.4(1.0-2.1)], respectively (Table 2). Younger participants had significantly optimal IQ compared to older participants although with weak effect size (t=10.8, df=1120, p<0.001, effect size= -0.1).

**Table 2:** the relationship between socio-demographic variables and intelligence quotient

Variables	Intelligence quotients		χ 2	p-value
	Optimal	Suboptimal		
**Gender**			1.6	0.20
Male (n=521)	292(56.0%)	229(44.0%)		
Female (n=601)	314(52.2%)	287(47.8%)		
**Social class**			84.4	<0.001*
Upper (n=439)	309(70.4%)	130(29.6%)		
Middle (n=300)	147(49.0%)	153(51.0%0		
Lower (n=383)	150(39.2%)	233(60.8%)		
**School type**			128.2	<0.001*
Public (n=517)	185(35.8%)	332(64.2%)		
Private (n=605)	421(69.6%)	184(30.4%)		
**Family size**			28.6	<0.001*
1-4 (n=631)	387(61.3%)	244(38.7%)		
>4 (n=476)	215(45.2%)	261(54.8%)		

**Relationship between socio-demographic variables and academic performance:** the association between socio-demographic characteristics and academic performance are shown in Table 3, Table 4, Table 5. There was a significant association between academic performance and gender (p=0.02) such that females were more likely than males to have higher academic performance AOR, (95% CI), 2.4(1.4-4.3)]. Being from upper social class (p<0.001), in private school (p=0.001), and family size less than 4 (p<0.001) were significantly associated with high academic performance (Table 6).

**Table 3:** the relationship between of socio-demographic variables and academic performance

Variables	Academic performance			χ 2	p-value
	Low	Average	High		
**Gender**				7.6	0.02*
Male (n=521)	38(7.3%)	197(37.8%)	286(54.7%)		
Female (n=601)	28(4.7%0	199(33.1%)	374(62.2%)		
**Social class**				138.7	<0.001*
Upper (n=439)	4(0.9%)	91(20.7%)	344(78.4%)		
Middle (n=300)	16(5.3%)	125(41.7%)	159(53.0%)		
Lower (n=383)	46(12.0%)	180(47.0%)	157(41.0%)		
**School type**				195.8	0.001*
Public (n=517)	56(10.8%)	270(52.2%)	191(36.9%)		
Private (n=605)	10(1.7%)	126(20.8%)	469(77.5%)		
**Family size**				41.4	<0.001*
1-4 (n=631)	30(4.8%)	174(27.6%)	427(67.7%)		
>4 (n=476)	33(6.9%)	212(44.5%)	231(48.5%)		

**Table 4:** logistic regression of the socio-demographic predictors of intelligent quotients

Socio-demographic variables	Crude Odd Ratio (95CI)	Adjusted Odd Ratio (95% CI)
**Social class**		
Lower versus upper*	3.7(2.8-4.9)	2.9(1.8-3.6)
Lower versus middle**	1.5(1.1-2.0)	1.1(0.9-3.9)
**School type**		
Public	4.1(3.2-5.3)	3.7(2.9-4.8)
Private		-
**Family size**		
≤4	-	-
>4	1.9(1.5-2.5)	1.4(1.0-2.1)

Dependent variable (intelligent quotient; 0=Suboptimal and 1=optimal); reference category *=upper class, **=middle class)

**Table 5:** multinomial logistic regression of the association between academic performance and some socio-demographic parameters

Variables	Academic performance	Coefficient	Wald	P-value	AOR	95%C.I
	High					
**Gender**						
Female		0.88	9.1	0.003	2.4 -	1.4-4.3 -
male		-	-	-		
**Social class**						
Upper		2.1	12.5	<0.001	8.1	7.8-51.6
Middle		0.6	3.2	0.07	1.9	0.9-3.8
Lower		-	-	-	-	-
**School type**						
Private		1.6	14.8	<0.001	5.1	2.2-11.5
Public		-	-	-	-	-
**Family size**						
1-4		-0.2	1.0	0.3	0.7	0.4-1.3
>4		-	-	-	-	-
	Average					
**Gender**						
Female		0.5	2.7	0.1	1.6	0.9-2.8
male		-	-	-	-	-
**Social class**						
Upper		1.7	7.6	0.006	5.2	1.6-16.8
Middle		0.8	5.1	0.02	2.2	1.1- 4.3
Lower		-	-	-	-	-
**School type**						
Private		0.5	1.2	0.27	1.6	0.7-3.7
Public		-	-	-	-	-
**Family size**						
≤4		-0.4	2.3	0.13	0.6	0.4-1.1
>4		-	-	-	-	-

Dependent variable= academic performance, where high=0, average=1, low=2; reference category = lower social class, public schools, and family size ≤4; AOR=Adjusted Odd Ratio

**Table 6:** the relationship between age, intelligence quotient and academic performance

Variables	Intelligence quotient		Test-stat	df	p-value	Effect size (95% CI)
	Optimal	Sub-optimal				
Academic scores	80.3±13.09	71.0±15.8	t=-4.1	1120	<0.001	1.0(0.4-1.5)
Age (years)	8.8±1.61	9.3±1.7	t=10.8	1120	<0.001	-0.1(-0.7-0.6)

## Discussion

The proportion of children with optimal IQ in this study were more than those with suboptimal IQ. This differed from the observations of similar studies which assessed intelligence using the same tool. Ejekwu *et al*. [12]. in Enugu, Adedeji *et al*. [13] in Jos and Ijarotimi *et al*. [23] in Akure noted suboptimal intelligence in 69.7%, 62.7% and 83.4% of their study participants respectively. These variations with the current study could be explained by difference in methodology. Ejekwu *et al*. [12]. studied only public schools. The exclusion of children in the private schools limits generalization of their result. Although Adedeji *et al*. [13] and Ijarotimi *et al*. [23] like the index study, studied both private and public schools in the urban cities of Jos and Akure respectively, a greater proportion of their study participants were stunted and wasted. Ninety-four percent of the children in this study had an academic performance of at least 50%. The fact that most of these children performed above average academically could be related to the high rate of optimal intellectual ability. However, unlike the intellectual assessment which used a uniform tool, the academic assessment was not uniform as it differed from school to school. As a result, bias cannot be excluded. Using past school records as the index study, Adeyemi [24] in Osun noted a below average academic performance of 9.2% in private primary schools while Abebe *et al*. [25] in Ethiopia noted 23.2% of the pupils were below average. The result of this study compares with these studies as the majority had above average academic performance.

This study showed a significant association between age and intelligence quotient. Those who had optimal intelligence were significantly younger than those with suboptimal IQ. Ejekwu *et al*. [12] also noted that younger age group (less than 10 years) performed significantly better on Raven´s test. It´s been suggested that younger children pay better attention to instructions which may be responsible for their better performance.8 On the contrary, Survana *et al*. [26] noted a positive correlation of increasing intelligence score with age. They however assessed IQ using Standford Binet and very young people have been noted to perform poorly on this test as they lack the sustained concentration needed to complete the text [27]. Conversely, this study found that older children had a significantly better academic performance. This trend was also reported by Acham *et al*. [28] in Uganda. Abebe *et al*. [25] in Ethiopia also noted that children in grade 4 and above had higher odds of performing above average in academic achievement. This can be explained by the fact that sustained attention which increases with age is essential for effective learning and younger children lack this sustained attention. The association between IQ, academic performance and socio-economic status was significant in this study with the middle and lower class more likely to have suboptimal IQ than those in the upper class. Their odds of performing sub-optimally were about two to three times that of the upper class. Adedeji *et al*. [13] and Ejekwu *et al*. [12] observed that lower socio-economic indices significantly affected intelligence scores while Abebe *et al*. [25] and Ampofo *et al*. [29] documented significant relationship between academic performance and lower socio-economic status.

The relationship between socio-economic class, intelligence quotient and academic performance may be mediated by a third factor. The lower IQ in lower socio-economic class is largely attributable to the unfavourable environmental factors that usually accompany poverty. Indices of social disadvantage such as the risk of diseases/illnesses for example iron deficiency anaemia, under-nutrition and low level of parental education are common among persons in the lower socio-economic and could lead to impaired cognitive function [13, 30]. For example, Asmare *et al*. [30], found that indicators of under-nutrition are significantly associated with reduced intelligence and academic performance. Although nutritional status assessment was outside the scope of the present paper, previous reports have shown a positive correlation with intelligence and academic performance [13, 30]. Another possible mediator of the relationship between socio-economic class, intelligence quotient and academic performance may be parental education. In calculating the child´s social class, parental education and occupation is considered. In other words, the finding that children from upper socio-economic class were more likely to have higher intelligence quotient and academic performance may be related to a third factor-parental education. Bakar *et al*. [31] reported that children from families where parents are less educated tend to perform systematically worse in school than pupils whose parents have more education. In addition, these children are more likely both at home and in schools to be deprived of surroundings that stimulate proper cognitive development.

The result of this study showed that children in public schools were more likely to perform sub-optimally in the test of intelligence and to have a low academic performance. This trend was also documented by Adedeji *et al*. [13], Ijarotimi *et al*. [23] and Millones *et al*. [32]. This could be explained by the more conducive learning atmosphere observed in private schools which encourage intellectual stimulation and development. Academic performance was significantly better among females. This is similar to the findings by Duckworth A *et al*. [33] and Abebe *et al*. [25]. Females spend more time indoors and have better opportunities to study contrary to the males who spend a lot of time outdoors playing with their friends [25]. Boys are prone to disruptive and inattentive classroom behaviours that retard learning leading to a male educational disadvantage. Girls are also said to be more obedient than boys. They pay more attention to details than boys and as a result, are said to learn better than boys. In addition, the differences in gender with respect to academic performance may be mediated by emotional intelligence [34]. Generally, there literature is inconsistent with regards of gender differences with emotional intelligence (EI), but some authors have reported better EI with females. Emotional intelligence has been shown to be associated with more pro-social behaviours and better academic performance. Although not examined in this study, this gender differences in academic performance may have been influenced by the EI.

## Conclusion

Age, low socio-economic status, large family size and public school attendance impact negatively on IQ and academic performance. Hence, measures to curb large family sizes and improve the socio-economic status of families should be put in place.

### What is known about this topic

- There has been differing reports in the proportion of school-aged children with optimal intelligent quotient, and most of the studies studied intelligence quotient only and concentrated only in the government owned schools;
- There has been no consensus on the relationship between intelligence quotient and academic performance. Some authors noted a positive correlation where high IQ was associated with high academic performance, while some found no correlation between the two.

### What this study adds

- This study has gone a step further to look at both intelligent quotient and academic performance in both government owned schools and private owned schools;
- Low socio-economic status, large family size and public school attendance impact negatively on IQ and academic performance;
- IQ and academic performance were positively correlated with each other.
